# IL-33 Is Produced by Mast Cells and Regulates IgE-Dependent Inflammation

**DOI:** 10.1371/journal.pone.0011944

**Published:** 2010-08-03

**Authors:** Chia-Lin Hsu, Colleen V. Neilsen, Paul J. Bryce

**Affiliations:** Division of Allergy-Immunology, Feinberg School of Medicine, Northwestern University, Chicago, Illinois, United States of America; New York University, United States of America

## Abstract

**Background:**

IL-33 is a recently characterized IL-1 family cytokine and found to be expressed in inflammatory diseases, including severe asthma and inflammatory bowl disease. Recombinant IL-33 has been shown to enhance Th2-associated immune responses and potently increase mast cell proliferation and cytokine production. While IL-33 is constitutively expressed in endothelial and epithelial cells, where it may function as a transcriptional regulator, cellular sources of IL-33 and its role in inflammation remain unclear.

**Methodology/Principal Findings:**

Here, we identify mast cells as IL-33 producing cells. IgE/antigen activation of bone marrow-derived mast cells or a murine mast cell line (MC/9) significantly enhanced IL-33. Conversely, recombinant IL-33 directly activated mast cells to produce several cytokines including IL-4, IL-5 and IL-6 but not IL-33. We show that expression of IL-33 in response to IgE-activation required calcium and that ionomycin was sufficient to induce IL-33. In vivo, peritoneal mast cells expressed IL-33 and IL-33 levels were significantly lower within the skin of mast cell deficient mice, compared to littermate controls. Local activation of mast cells promotes edema, followed by the recruitment of inflammatory cells. We demonstrate using passive cutaneous anaphylaxis, a mast cell-dependent model, that deficiency in ST2 or antibody blockage of ST2 or IL-33 ablated the late phase inflammatory response but that the immediate phase response was unaffected. IL-33 levels in the skin were significantly elevated only during the late phase.

**Conclusions/Significance:**

Our findings demonstrate that mast cells produce IL-33 after IgE-mediated activation and that the IL-33/ST2 pathway is critical for the progression of IgE-dependent inflammation.

## Introduction

Mast cells are important in both innate and adaptive responses and best characterized for their roles in defense against invading pathogens and hypersensitivity responses. Activation of mast cells leads to the release of potent inflammatory mediators, including preformed mediators (e.g. histamine), lipid metabolites (e.g. prostaglandins) and a plethora of cytokines and chemokines [1]. Mast cells can also be activated by a variety of stimuli and release distinct patterns of mediators, depending on the type and strength of stimuli [2]. The most characterized pathway to mast cell activation is antigen-mediated crosslinking of IgE molecules that bind via FcεRI [3], that is highly expressed on mast cells and important in allergic diseases.

Interleukin-33 (IL-1F11, NF-HEV) is a newly characterized cytokine belonging to the IL-1 cytokine family that also includes IL-1α, IL-1β, IL-1Ra (IL-1 receptor antagonist) and IL-18 [4]. It contains an N-terminal predicted helix-turn-helix (HTH) motif responsible for nuclear translocation and chromatin binding [5] and an IL-1-like C-terminal domain. Full-length IL-33 is about 30 kDa and early data suggested that IL-33 required cleavage by caspase-1 at Ser_111_ to be biologically active (about 18 kDa) [4]. However, more recent studies have defined that full-length IL-33 is biologically active [6], [7], [8]. While Schmitz et al. proposed that the cleavage site for activation was at Ser_111_ [4], Cayrol and Girard have more recently demonstrated cleavage within the IL-1-like C-terminal domain at Asp_178_ that inactivates IL-33 [6].

IL-33 exerts its function by binding on its receptor ST2 (T1/ST2, Fit-1, DER4), in associated with IL-1AcP and, in several studies utilizing a recombinant 18 kD IL-33 molecule (representing the predicted Ser_111_ cleaved product (aa109-266 for mouse, aa112-270 for human)), has been shown to modulate parasite expulsion [9], pain sensitivity to antigen stimulation [10], experimentally induced atherosclerosis [11], induce cutaneous fibrosis [12], promote recruitment of Th2 cells, basophils and eosinophils [13], [14], as well as potently induce the activation and survival of mast cells, independently of FcεRI [15], [16], [17]. It also enhances atypical Th2 response by promoting IL-5 and IL-13 production by T cells [18]. Recent data has also shown that in vivo administration of IL-33_109–266_ can enhance IgE-driven anaphylactic shock [19].

Despite this wealth of data, demonstrating exogenous IL-33_109–266_ as an inflammatory regulator, the sources and functions of IL-33 in vivo remain less characterized. This is despite mounting evidence for IL-33 expression in type 2 inflammatory diseases, including severe asthma [20], allergic conjunctivitis [21] and inflammatory bowel disease [22]. IL-33 protein is expressed by high endothelial venule cells, in which it functions as a transcriptional regulator [5], much like IL-1α and IL-18 have been shown to do [23], [24], and has also been found within the nucleus of epithelial cells [25]. The initial profiling of organs or cells for IL-33 mRNA demonstrated expression in purified dendritic cells, epithelial cells, activated macrophages and, interestingly, high expression in stomach, lung, brain and skin tissues [4], all sites that are rich in mast cells. Additionally, adipocytes have also been shown to express mRNA for IL-33 [26] however their ability to produce IL-33 protein is unknown. This may be an important consideration since skin fibroblasts, which were initially shown to express IL-33 mRNA [4], have since been shown to have no detectable IL-33 protein [25]. A recent report showed that IL-33 can be expressed and released by activated macrophages independent on caspase-1, -8 and calpain [27]. However, the specific sources of IL-33 protein during Th2-type inflammation remain poorly defined.

We hypothesized that mast cells might be a source of IL-33 due to their importance in a diverse range of inflammatory responses, and particularly Th2-type immunity. Mast cells also express the IL-33-related family members IL-1β and IL-18 upon their activation [28], [29]. However, unlike IL-1β and IL-18, IL-33 specifically promotes Th2-associated responses [4], [30], which mast cell activation has also been shown to do [31]. The results presented here demonstrate that mast cells produce IL-33 and that IgE-mediated activation potently enhances expression, both in vitro and in vivo. Interestingly, IL-33 fails to induce its own expression and we show a requirement for calcium-mediated processes in IL-33 expression. Functionally, we demonstrate that the IL-33/ST2 axis is important for the late phase response in a mast cell-dependent model of anaphylaxis by recruiting of inflammatory cells into the site of mast cell activation. Therefore, our study defines IL-33 as an important mediator in regulating the events between IgE-mediated mast cell triggering and the development of subsequent inflammatory responses.

## Materials and Methods

### Reagents

Rabbit polyclonal anti-mouse IL-33 and recombinant IL-33_109–266_ were obtained from Axxora. Mouse IgE (SPE-7 clone), dinitrophenyl (DNP)-human serum albumin (DNP-HSA) and ionomycin were obtained from Sigma. Murine IL-33 Quantikine ELISA kit, rat monoclonal anti-mouse IL-33, rat IgG2a isotype, goat anti-mouse ST2 for PCA model, goat IgG isotype and mouse ST2/IL-1 R4 DouSet ELISA were from R&D Systems. Taqman gene expression primers and probes were obtained from Applied Biosystems. Murine OVA-specific IgE was produced from the TOε hybridoma, as previously described [32]. Anti-CD16/32 (2.4G2), anti-CD117 (c-kit), anti-FcεRI (MAR-1) were obtained from BD Biosciences. Rat anti-mouse ST2 for flow cytometry was obtained from MDBiosciences.

### Animals

BALB/c and C57BL/6 female mice (4–6 weeks) were obtained from Taconic Farms. WBB6F1/J-*Kit^w^/Kit^Wv^* (W/Wv) mice and wildtype littermates (4–6 weeks) were obtained from Jackson Labs. ST2 deficient mice were generated as previously described [33] and obtained from Dr Andrew McKenzie at the MRC Laboratory of Molecular Biology, Cambridge. Mast cell reconstitution of W/Wv mice was performed by injection of 1×10^7^ BMMC and determined by toluidine blue staining of tissues after 12 weeks while hematocrits were performed to confirm no influence on anemia.

### Ethics Statement

All animal studies were performed under the strict guidelines for care and welfare set out by IACUC and in accordance with animal protocol ID 2007-1260, which has been reviewed and approved by the Northwestern University Animal Care and Use Committee.

### Mast cell cultures

Bone marrow-derived mast cells (BMMC) were obtained by flushing bone marrow from femurs using complete RPMI media, 10% FBS, penicillin/streptomycin and L-glutamine. Cells were then cultured in mast cell growth media (complete RPMI media +30 ng/ml recombinant mouse IL-3) for 5 weeks. Mast cell phenotype was determined by toluidine blue straining (>96%) and flow cytometry for double stained c-kit^+^/IgE binding^+^ cells (>95%). MC/9 cells were obtained via the American Tissue Culture Collection and maintained in mast cell growth media.

### Gene Expression Analysis

Expression of mRNA for murine IL-4, IL-5, IL-6, IL-33 and ST2 were determined using commercially available FAM-labeled Taqman primers and probes (Applied Biosystems) on cDNA derived from mRNA extracted using a Qiagen RNeasy mRNA isolation kit. Gene expression was determined based on the delta CT values between gene of interest and β-actin and compared to the mean delta CT values for the control group to determine a fold induction value, as previously described [34].

### Flow cytometry

For intracellular staining of IL-33, unprimed or IgE primed BMMC cells were stimulated for 24 hours with 1 µM ionomycin or 0.5 µg/ml DNP-HSA in mast cell growth media. Peritoneal cells were harvested by lavage of the peritoneal cavity with PBS. Cells were blocked with anti-CD16/32 and permeabilized using BD Cytoperm/wash (BD Pharmingen). Cells were stained for 1 hour using anti-IL-33 (2 µg/10^6^ cells), followed by anti-rabbit PE-labeled secondary antibody. Peritoneal cells were also stained with CD117 (c-kit) and FcεRI to enable identification of mast cells. For surface staining of ST2, unprimed or IgE primed BMMC cells were stimulated for 6 hours with or without 0.5 ug/ml DNP-HSA in mast cell growth media. Cells were blocked with anti-CD16/32 and stained for 1 hour using rat monoclonal anti-ST2 (1 µg/10^6^ cells), followed by anti-rat FITC-labeled secondary antibody.

### Western Blot

Unprimed or IgE primed BMMC cells were stimulated for 24 hours with 0.5 µg/ml DNP-HSA in mast cell growth media. Cells were lysed under sonication and 100 µg of total protein, determined by Bradford Assay (Bio-Rad), was separated by 10% SDS-PAGE and transferred to nitrocellulose membrane. Membranes were probed with polyclonal rabbit anti-mouse IL-33 (1∶2000). Membranes were stripped and reprobed for β-actin to confirm equal loading.

### Tissue Cytokine Quantification

Ear skin was homogenized in phosphate buffered saline (PBS) containing 1∶100 dilution of protease inhibitor cocktail (Sigma) and the supernatant collected. The quantity of IL-33 in the supernatant was determined using a murine IL-33 Quantikine ELISA (R&D Systems) and normalized to the total protein content of each sample, determined by BCA assay (Invitrogen).

### Passive Cutaneous Anaphylaxis (PCA) and Immunohistochemistry

PCA was performed as previously described [35] using intradermal anti-DNP IgE (100 ng) (SPE-7 clone, Sigma) and intravenous DNP-HSA (100 µg). Where indicated, anti-ST2 or IL-33 was administrated 1 hour before DNP-HSA challenge. The thickness of ears was measured at the indicated time points. In some experiments, ear tissue was collected 24 hours after intravenous challenge, frozen and embedded in OCT compound. 7 µm sections were cut and fixed with ice cold 1∶1 acetone/methanol before staining using a DAB- immunohistochemistry detection kit (R&D systems), as per the manufactures instructions. Rabbit polyclonal anti-IL-33 (Axxora) (1∶500) was used as a primary detection antibody and biotinylated goat anti-rabbit IgG (Zymed) was used as a secondary antibody. After staining, sections were counterstained with Mayer's hematoxylin. Images were generated using a Leica spinning-disc confocal microscope and Spot-image analysis software.

## Results

### Bone Marrow-derived Mast Cells Express IL-33 Upon IgE-driven Activation

**Figure 1:**
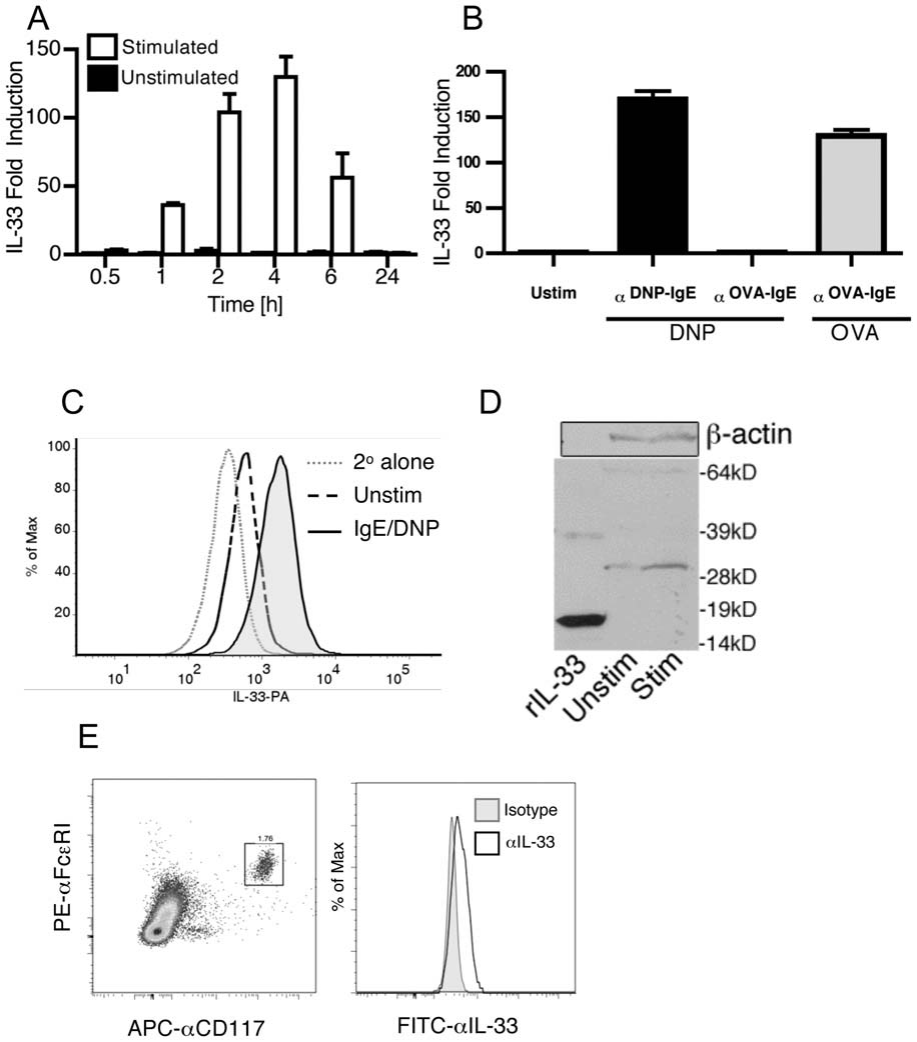
In vitro mast cells produce IL-33 upon specific activation by IgE. 1×10^6^ BMMC were primed with 1 µg/ml anti-DNP IgE (SPE-7 clone) overnight and stimulated by addition of 0.5 µg/ml DNP-HSA (black bars) or vehicle control (white bars). After the indicated times, gene expression of mouse IL-33 was determined by real-time RT-PCR (A). Either anti-DNP IgE or anti-OVA IgE was used in combination with either DNP-HSA or OVA, as indicated, and IL-33 gene expression determined (B). Intracellular staining for IL-33 was determined in permeabilized BMMC 24 hours after stimulation of 0.5 µg/ml DNP-HSA (C). Western blot analysis of BMMC lysate from stimulated or unstimulated cells 24 hours after stimulation (D). Recombinant, mouse IL-33_109–266_ was used as a positive control. Data represents 3 independent experiments. Primary mast cells were obtained by peritoneal lavage, identified as CD117^+^/FcεRI^+^ and intracellular staining for IL-33 performed after permeabilization (E). Data represents 2 independent experiments.

We initially investigated if mast cells could also produce IL-33. Utilizing DNP-specific IgE primed BMMC, we show that IL-33 mRNA was upregulated within 30 minutes of stimulation and peaked at 4 hours after antigen-driven activation with DNP-HSA (Figure 1A). While IL-1β and IL-18 were also increased (data not shown), the expression of IL-33 was more than ten fold higher than either of these related cytokines. This was antigen specific, since DNP-HSA stimulated expression of IL-33 in mast cells primed with DNP-specific IgE but not OVA-specific IgE while concomitantly, OVA induced expression in these cells (Figure 1B). In addition, we determined whether IL-33 protein could be detected within mast cells by flow cytometry. We found that IL-33 protein was detectable in unstimulated cells and enhanced in IgE/DNP stimulated cells (Figure 1C). We then investigated if mast cell-expressed IL-33 had undergone cleavage or not. Low levels of IL-33 protein were detected in unstimulated cells at a full-length size of 31 kD and IgE-mediated stimulation upregulated this form, with no evidence of cleavage products (Figure 1D). Quantification of three independent experiments demonstrated a significant increase in the relative band intensity of the IL-33 protein (858±121 in unstimulated cells to 1393±250 in stimulated cells). ATP addition (10–500 µM), which has been demonstrated to promote caspase-1-activated IL-1β secretion [36], was insufficient to induce secretion of IL-33 (data not shown), supporting the lack of a caspase-1 mediated release mechanism for IL-33, while LPS (5–100 ng/ml), shown to promote release of IL-6 and TNFα from BMMC [37], had no effect on release either with or without concurrent IgE-mediated stimulation (data not shown). Finally, we utilized intracellular staining to investigate if primary mast cells also expressed IL-33. Peritoneal lavage was performed and the recovered cells contained a distinct population of CD117^+^/FcεRI^+^ mast cells that stained positive for IL-33 (Figure 1E).

### IL-33 expression by mast cells is dependent on calcium and not induced by IL-33

**Figure 2:**
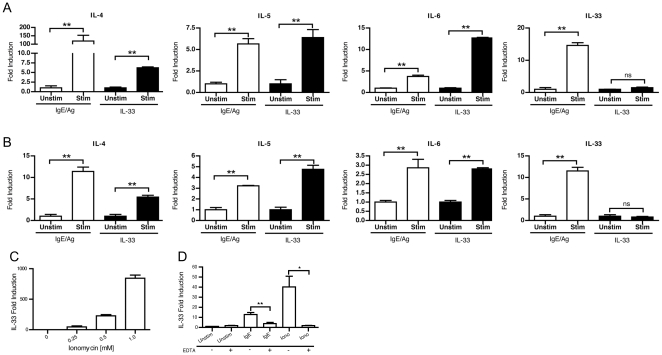
Mast cell expression of IL-33 requires calcium and is not induced by IL-33. 1×10^6^ BMMC were primed with 1 µg/ml anti-DNP IgE (SPE-7 clone) overnight and stimulated by addition of 0.5 µg/ml DNP-HSA or 10 ng/ml of recombinant IL-33_109–266_. After 4 hours, the gene expression of cytokines was determined by real-time RT-PCR in BMMC (A) and in MC/9 cells (B). IL-33 expression in response to ionomycin stimulation was determined using BMMC (C). The expression of IL-33 was then determined in BMMC after stimulation with 0.25 µM ionomycin or IgE/antigen in the presence or absence of 5 mM EDTA (D). * = p<0.05, ** = p<0.01 by Students *t*-test. Data represents the mean±SEM from 6 individual wells over two independent experiments.

We further explored the regulation of IL-33 expression in mast cells. IL-33 itself has been shown to be a potent inducer of mast cell cytokine production [15], [16], [17], [38], independent of FcεRI signaling. We postulated that released IL-33 might promote its own expression by activating mast cells, in a potentially autocrine fashion. To test this, we compared the expression of cytokine genes in response to IgE/FcεRI activation or to IL-33 activation. The expression of IL-4, IL-5, IL-6 and IL-33 were induced in BMMC (Figure 2A) after either stimulation. However, IL-33 was uniquely stimulated by IgE-mediated activation and not by IL-33. Similar results were obtained using MC/9 cells, a murine mast cell line (Figure 2B). We next compared the signaling pathways from FcεRI and ST2. The pathways for IgE-mediated FcεRI activation are well characterized and calcium-mediated activation is vital for many downstream events [3]. Conversely, IL-33 mediated activation via ST2 and the IL-1 receptor accessory protein (IL-1RAcP) activates ERK, JNK, p38, IκB and NF-κB [4], [39]. Therefore, we investigated the role of calcium processes in the expression of IL-33 in mast cells. Ionomycin (0.25–1 µM) treatment of BMMC was sufficient to induce robust, dose-dependent expression of IL-33 (Figure 2C) as well as protein expression by intracellular staining (data not shown). As shown in Figure 2D, ionomycin-induced IL-33 could be suppressed by addition of EDTA (5 mM) into the culture media. Similarly, EDTA suppressed the expression of IL-33 by IgE-mediated activation. Together, these data demonstrate that the expression of IL-33 by mast cells is dependent upon calcium activation, such as occurs via IgE/FcεRI but not via ST2.

### IgE/antigen or ionomycin stimulation alters ST2 expression on mast cells

**Figure 3:**
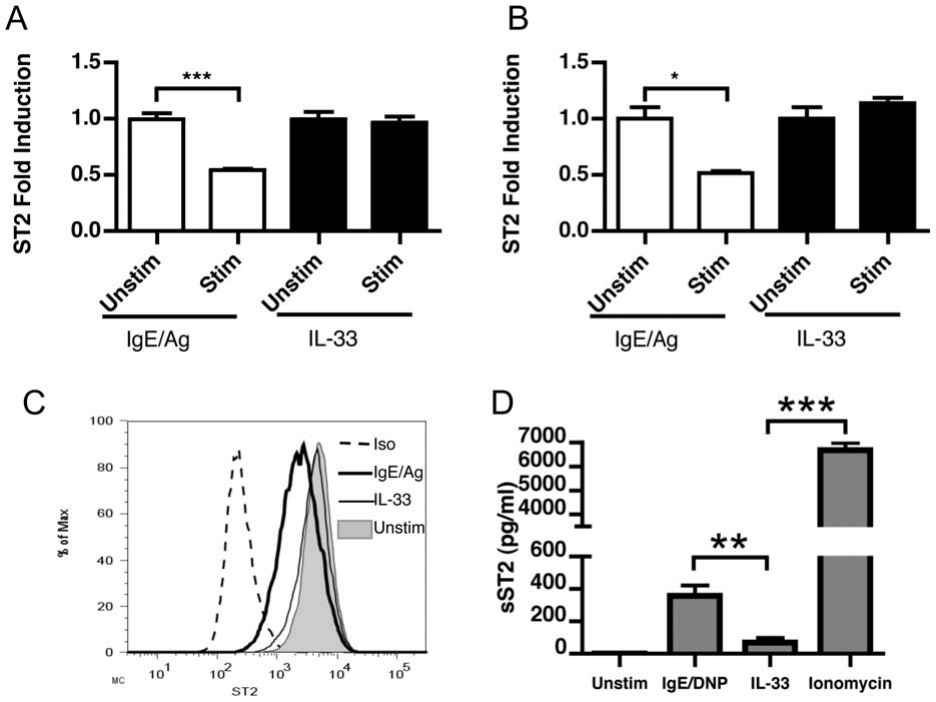
ST2 is downregulated on mast cells after IgE-driven activation. 1×10^6^ BMMC (A) or MC/9 cells (B) were primed with 1 µg/ml anti-DNP IgE (SPE-7 clone) overnight and stimulated by addition of 0.5 µg/ml DNP-HSA or 10 ng/ml of recombinant IL-33_109–266_. After 4 hours, the gene expression of ST2 was determined by real-time RT-PCR. Surface expression of ST2 protein on BMMC was determined by flow cytometry after 6 hours (C). The levels of ST2 in supernatants were analyzed by ELISA after 6 hours (D). * = p<0.05, *** = p<0.005 by Students *t*-test. Data in A and C represents the mean±SEM from 3 to 6 individual wells over two independent experiments. Data in B is representative of results from three independent experiments.

Interestingly, we also found IgE-dependent activation induced a significant decrease in the mRNA for ST2 within BMMC (Figure 3A) and MC/9 cells (Figure 3B). Conversely, IL-33 activation did not alter ST2 expression. In addition, the fold induction of the co-receptor IL-1RAcP was significantly reduced (1.00±0.03 for unstimulated (n = 13), verus 0.33±0.03 for IgE/DNP stimulated (n = 10), p<0.05 by Students *t*-test). The surface levels of ST2 were also markedly reduced by activation via IgE but not by IL-33 (Figure 3C). Using a sST2 specific ELISA, supernatants of BMMC activated by IgE/DNP or IL-33 for 6 hours were analyzed to determine if ST2 was being shed from mast cells. IgE/DNP activated mast cells released significantly more sST2 into the supernatant sST2 than those from the IL-33 activated group (Figure 3D). We also saw a similar increase in supernatant sST2 by BMMC activated by 1 µM ionomycin (Figure 3D) and downregulation of ST2 surface expression (data not shown).

### Mast cells express IL-33 constitutively and upon activation in vivo

**Figure 4:**
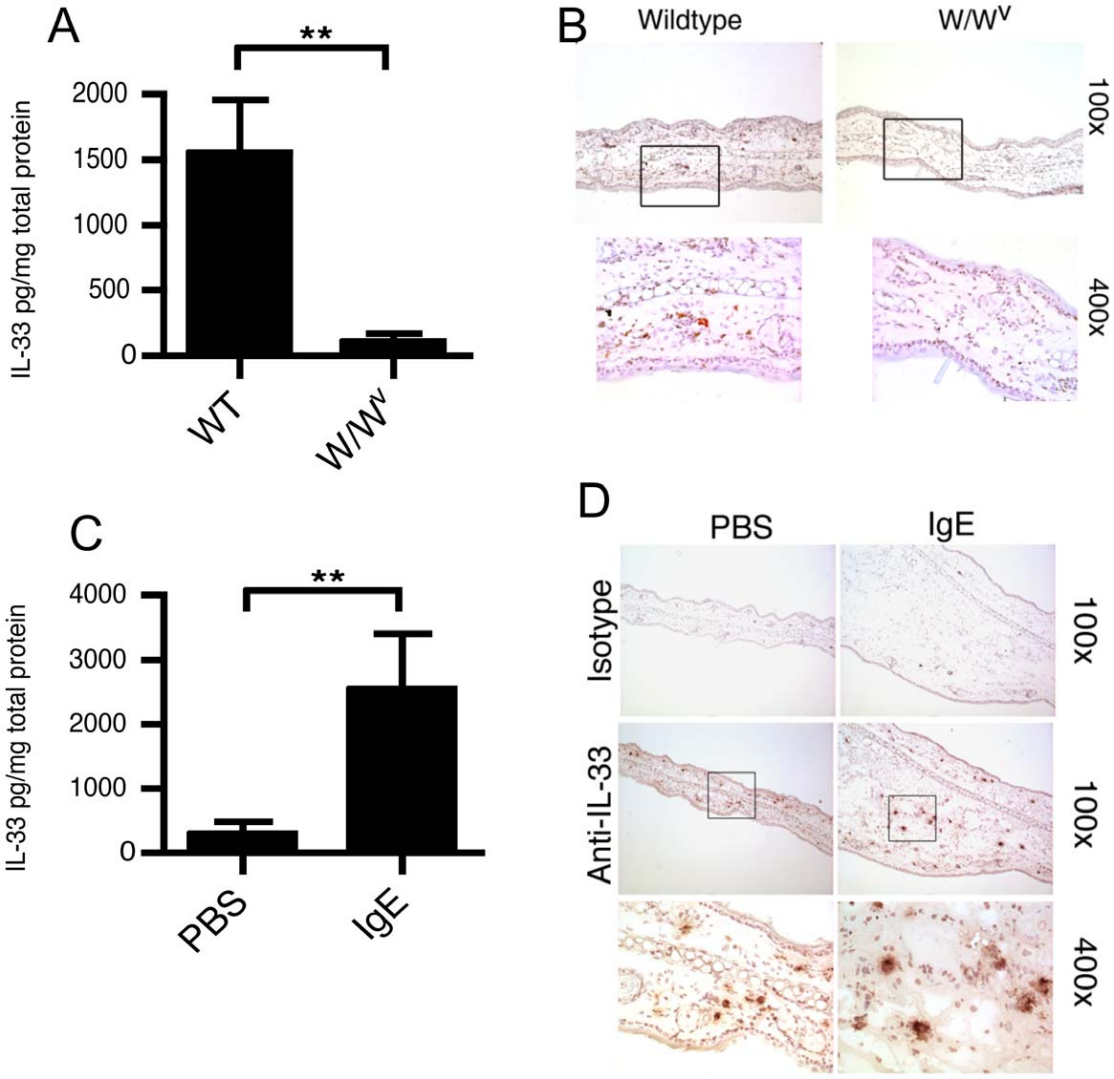
In vivo mast cells constitutively express IL-33 and increase expression during anaphylaxis. The tissue levels of IL-33 in the skin of wild type or mast cell deficient mice (W/Wv) were determined by ELISA (A). Additionally, frozen sections (7 µm) were stained for IL-33 immunoreactivity using biotinylated anti-IL-33 and ABC-DAB substrate and sections counterstained with hematoxylin (B). Total tissue IL-33 was determined in IgE-primed or sham skin in passive cutaneous anaphylaxis (C) and IL-33 immunoreactivity determined, as before (D). ** = p<0.01 by Students *t*-test. Data in panels A and C represents the mean±SEM from 5 individual mice while panels B and D are representative of results from 4 individual mice.

To test if mast cells are a source of IL-33 in vivo, we initially studied the levels of IL-33 within the skin of mast cell-deficient mice (Strain WBB6F1/J-*Kit^W^/Kit^W-v^*) or littermate controls. IL-33 was significantly lower in the skin of mast cell-deficient mice (Figure 4A) and was observed to be expressed mainly in the epidermal and dermal regions of control mice but absent in mast-cell deficient, as shown by immunohistochemistry (Figure 4B). To test if IL-33 was upregulated by IgE-driven activation, we investigated IL-33 levels in the skin using a passive cutaneous anaphylaxis model, whereby local mast cell activation is driven by passive IgE priming, followed by systemic antigen challenge. This model has been shown to be strongly mast cell-dependent [40], since mast cell-deficient mice fail to elicit responses but reconstitution of mast cells to the skin restores responses to control levels, as demonstrated in Figure S1. Figure 4C shows that IL-33 levels were significantly elevated at IgE-primed skin sites versus the sham (PBS) injected skin of the same mouse. Immunohistochemical staining for IL-33 showed small, compact-staining cells throughout the epidermal/dermal zones of sham treated skin (Figure 4D). However, IgE-treated skin demonstrated a significantly increased number of IL-33^+^ cells (7.2±0.36 positive cells per high powered field (hpf) for control skin versus 10.4±0.78 for IgE-treated, based on the average frequency in 10 hpf/animal, p<0.001 by Students *t*-test).

### IgE-mediated late phase inflammation is IL-33 and ST2 dependent

**Figure 5:**
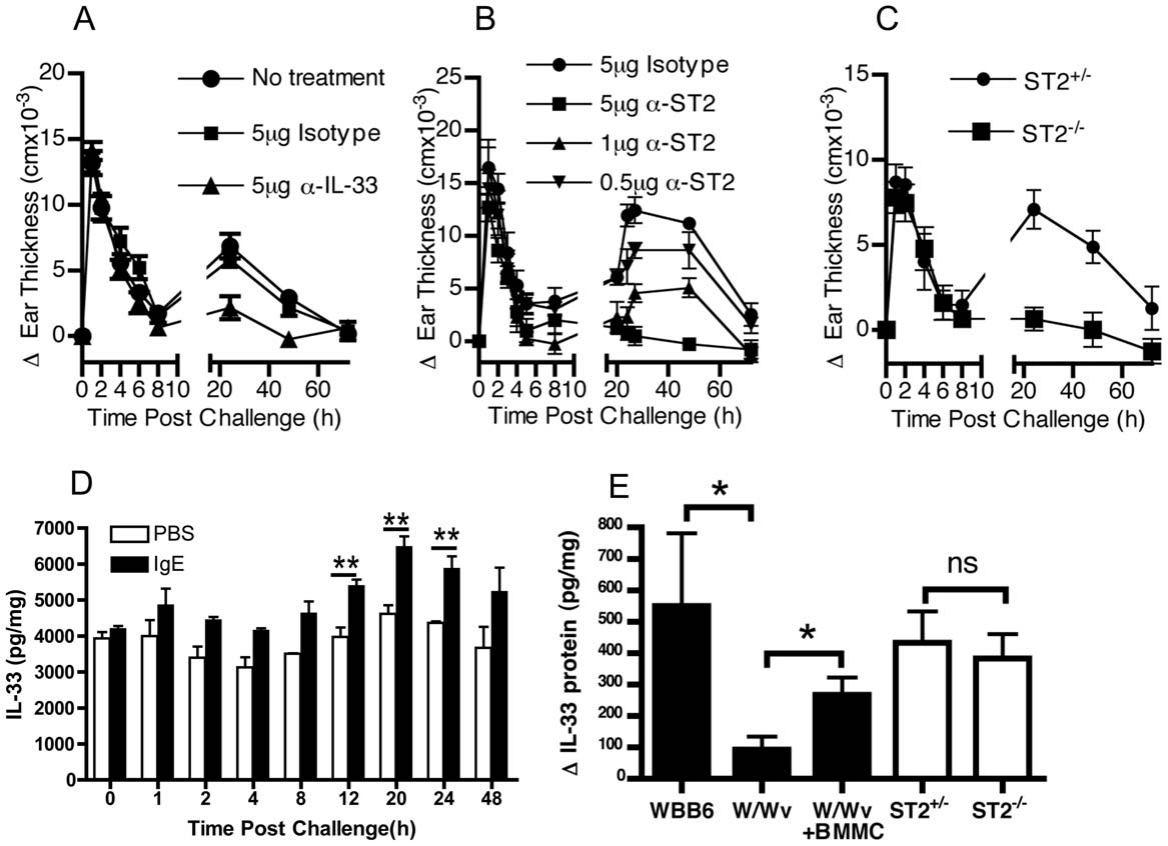
IL-33/ST2 pathway is critical for the development of IgE-driven tissue inflammation during anaphylaxis. Induction of ear swelling during passive cutaneous anaphylaxis was determined after systemic antigen challenge in mice receiving no treatment, 10 µg anti-murine IL-33 antibody or 10 µg isotype control (A), anti-murine ST2 antibody or isotype control (B) or in ST2^−/−^ or ST2^−/+^ control mice (C). Data represents the mean±SEM from 10–15 mice. The IL-33 protein levels in ear tissue from IgE injected or PBS injected sites were determined by ELISA during the PCA time course (D) (n = 3 mice per time point). Increases in IL-33 at IgE injected skin sites (determined as the delta between the PBS injected skin from the same individual) were determined in W/Wv, reconstituted W/Wv, ST2^−/−^ or relevant control strains (n = 3–9 mice per group). * = p<0.05, ** = p<0.01, *** = p<0.005 by Students *t*-test.

To investigate the functional effects of mast cell-derived IL-33, we utilized neutralizing antibodies against ST2 or IL-33 and ST2^−/−^ mice in the passive cutaneous anaphylaxis model. Figure 5A demonstrates that the initial wave of local tissue swelling, occurring with the first few hours after antigen challenge was unaffected by the administration of anti-IL-33 antibodies. However, mice treated with 5 µg of α-IL-33 showed a significantly reduced late phase response compared to the no treatment or isotype control groups (p<0.05 by 2-way ANOVA analysis). In addition, administration of neutralizing α-ST2 antibodies showed a similar diminution in the development of the late phase response (p<0.005 for Isotype versus 5 µg α-ST2, p<0.01 for Isotype versus 1 µg α-ST2 by 2-way ANOVA analysis) but no effect on acute response. ST2^−/−^ mice also had a similar early increase in ear swelling within the first few hours to their ST2^+/−^ littermates but lacked the late phase inflammation (Figure 5C).

Next, we investigated the temporal expression of IL-33 after initiation of anaphylaxis. Figure 5D demonstrates that IL-33 levels increased during anaphylaxis but was significantly elevated only during the late phase (12–48 hours) and not during the early phase (1–8 hours), when compared to either IgE primed skin collected prior to challenge (not shown) or when compared to time-matched PBS control skin (shown). Conversely, PBS injected skin showed no significant changes in IL-33 content at any time point. Finally, we determined the upregulation of IL-33 at 24 hours after antigen challenge in W/Wv, littermate controls or in W/Wv that were reconstituted with BMMC, as well as ST2^+/−^ and ST2^−/−^. IL-33 expression during anaphylaxis was significantly lower in W/Wv mice than controls while mast cell restoration led to a significant restoration (Figure 5E). Conversely, ST2^+/−^ and ST2^−/−^ demonstrated enhanced IL-33 expression to a similar degree, despite exhibiting differences in the generation of the late-phase inflammation (Figure 5C).

## Discussion

The results of our study demonstrate that mast cells express IL-33 and that IL-33 and ST2 are critical for the development of late-phase inflammation occurring during anaphylaxis. BMMC have basal expression of IL-33 and mast cell-deficient mice have significantly lower IL-33 within their skin. Upon activation, IgE/antigen stimulation promotes IL-33 expression via a calcium dependent mechanism that does not occur in response to IL-33-driven stimulation. To the best of our knowledge, these findings are the first demonstration of a functional effect of endogenous IL-33 in inflammation and indicate that IL-33 may be an important mechanism through which mast cells influence the recruitment and activation of inflammatory cells into sites of IgE-dependent activation.

IL-33 is being increasingly recognized as an important inflammatory cytokine. In particular, it exerts potent effects on several cell types involved in Th2-associated inflammation, including activation and migration of Th2 lymphocytes [4], [13], eosinophils [12], [41] and basophils [14], [30], [41]. High expression has also been found in Th2-associated diseases [20], [21], [22]. However, much of the current functional studies on IL-33 have relied upon administration of recombinant IL-33 and relatively little is known about endogenous production. While endothelial and epithelial cells express IL-33 constitutively, IL-33 was actually down-regulated in response to pro-inflammatory stimuli [42], supporting its suggested role as a transcriptional repressor. Our data clearly demonstrates that mast cells express IL-33 both in vivo and in vitro and that IL-33 is upregulated upon activation, indicating that mast cells may be an important source of IL-33 during inflammation, likely via its roles as a cytokine.

Mast cells are being increasingly recognized as critical cells in regulating a broad spectrum of immune responses, both protective and pathogenic. An important element in their abilities to influence immunity is their capacity to release a diverse panel of mediators, both preformed and de novo, and their ability to respond to many stimuli. Mast cell mediators are important in innate immunity, the transition to adaptive immunity and its strength of response, as well as the resolution of inflammation [43]. While activated mast cells are known to promote Th2-associated immunity [31], the critical mediators have remained elusive. IL-33 itself is amongst the spectrum of stimuli that can activate mast cells [15], [16], [17], [38]. Recently, using semi-quantitative approaches, Ohno et al. proposed that BMMC can express low levels of IL-33 mRNA in response to very high doses (30–100 ng) of IL-33 [27]. Our quantitative data shows that the expression of IL-33 by mast cells occurs in response to IgE-mediated activation but not to IL-33 concentrations that potently activated production of other cytokines, suggesting that autocrine amplification is unlikey. This is in contrast to several other cytokines, including IL-4 and IL-5, which have been shown to promote their own expression in mast cells [44], [45]. However, antigen-driven activation via crosslinking of IgE molecules, via FcεRI [46], is the critical pathway for the generation of immunological responses during anaphylaxis. That different stimuli, which drive similar expression of other cytokines, exhibit differential effects on IL-33 suggests that IL-33 might be an important determinate of the composition and strength of the inflammatory response.

One caveat to our in vitro studies using BMMC is that we have failed to detect secretion of IL-33 into the supernatants. It has been shown that BMMC represent a relatively immature phenotype and have lower expression of several co-stimulatory receptors, such as Toll-like receptors [47]. As such, BMMC may be functionally refractory to release of IL-33. However, fetal skin-derived mast cells, which express a more diverse range of Toll-like receptors [47], also failed to release IL-33 (data not shown). The mechanisms controlling release of IL-33 are unclear at this time. Recently, IL-33 was suggested to be produced by macrophages [27]. However, they also failed to detect any IL-33 in cell supernatants, suggesting that release of IL-33 is more complicated than a simple secretion process. Currently, only induction of necrosis has been shown to secrete IL-33 [7], leading to the proposal that IL-33 functions as an alarmin [48]. Determining the mechanisms through which IL-33 becomes extracellular is likely to be an important area for future studies on this cytokine. However, while our data failed to detect IL-33 release from mast cells in vitro, our passive cutaneous anaphylaxis experiments demonstrates a functional role for IL-33 and ST2 during in vivo inflammation. One possibility is that additional signals might be required for the secretion of IL-33 from mast cells. Mast cell-fibroblast interactions have been shown to be required for BMMC to secrete CCL11 (eotaxin-1), mediated via transmembrane expression of stem cell factor (SCF) by the fibroblast [49]. Similar cell-to-cell interactions might also be needed for mast cells to release IL-33. Whether IL-33 associated production and release processes are unique to mast cells or shared by other IL-33 expressing cells remains to be determined. Interestingly, we have observed differences in the baseline levels of IL-33 within the skin of the different strains of mice studied and yet the changes in the levels of IL-33 induced during anaphylaxis were relatively similar. This might suggest strain-specific influences on homeostatic IL-33 levels in cells such as endothelium that do not impact mast cell-derived IL-33.

The late phase of anaphylaxis is characterized by a robust recruitment of inflammatory cells but the mechanisms that regulate this process are poorly defined. In both the skin [40] and lung [50], antigen challenge of mice passively sensitized to antigen-specific IgE promoted a late-phase inflammatory response. Kaneko et al. demonstrated that the activation of mast cells promotes late-phase anaphylactic neutrophil, but not eosinophilic, accumulation [51] in the peritoneal cavity also. We recapitulated in our own studies that this is highly mast cell dependent, as previously demonstrated [40]. Both early and late-phase responses fail to occur if mast cells lack IκB kinase (IKK) 2 [52], disrupting IgE-mediated activation. TNF has been described as regulating late-phase inflammation [40], perhaps via phospholipase A2 activation [53], but this effect is only partial. Our data now demonstrates that IL-33 is a critical cytokine in regulating the late-phase response. Despite observing some differences in the magnitude of responses generated between experiments, in all cases interrupting the IL-33/ST2 axis, through the use using anti-IL-33, anti-ST2 or ST2 deficient mice, ablated this response. We also demonstrate that deficiency in ST2 does not alter the expression of IL-33 during anaphylaxis, suggesting ST2 is regulating responses distal to the expression of IL-33 rather than acting as an autocrine enhancer of mast cell activation. Interestingly, during such late-phase responses, the cellular influx is predominantly neutrophilic and neutrophils have been described to not express high levels of ST2 or respond to IL-33 [54]. However, neutrophils and monocytes are actively recruited to sites of basophil-mediated inflammation [55]. Recently, IL-33 has been shown to enhance basophils migration in vitro in response to CCL11 [14]. Therefore, mast cells could trigger the recruitment of late-phase inflammation via IL-33-mediated recruitment of ST2-expressing basophils to the site of antigen exposure. A second possible mechanism is that IL-33 and ST2 are required to enhance mast cell production of TNF, or other mediators, and IL-33 does enhance mast cell production of TNF [38].

Intriguingly, a study recently showed that IL-33 is markedly elevated in patients during anaphylactic shock, suggesting IL-33 may also play a potential role in IgE-mediated reactions in humans also [19]. Recombinant IL-33 has been shown to enhance mast cell responses in murine anaphylactic shock and these authors concluded that IL-33 enhances mast cell reactivity to IgE stimulation [19]. However, our data demonstrates that IgE-mediated activation is associated with reduction of ST2 expression and an increased release of sST2. Therefore, while IL-33 may be sufficient to enhance IgE-mediated mast cell activation, we propose that IgE-mediated activation might actually reduce the capacity of that mast cell to subsequently respond to IL-33.

In conclusion, our data defines mast cells as a novel endogenous source of IL-33. This occurs in response to IgE-mediated activation but not to IL-33-driven activation and we believe that this is likely due to the lack of calcium activation from ST2, compared to FcεRI, and we demonstrate that calcium is both necessary and sufficient for IL-33 expression by mast cells. Functionally, we show that IL-33 is expressed within the skin and is upregulated during IgE-driven anaphylaxis. This expression correlates with late-phase inflammation, rather than early phase reactions and both IL-33 and ST2 are dispensable for the early phase response during anaphylaxis but are required to elicit a late phase inflammatory reaction, a mast cell-dependent process that has been undefined to date. Mast cells are important regulators of both innate and adaptive immunity and we postulate that mast cell-derived IL-33 may be an important mediator in signaling the recruitment of inflammatory cells into peripheral tissues and their subsequent activation.

## Supporting Information

Figure S1 Passive cutaneous anaphylaxis is a mast cell-dependent model. Mast cells deficient (W/Wv) mice with or without reconstitution of mast cells with BMMC and littermates (WT) were investigated for their responses using the PCA model, as described in the methods. Ear swelling was measured at the indicated times.
(0.14 MB TIF)
