# Supplementary figures and images for: IL-33 Is Produced by Mast Cells and Regulates IgE-Dependent Inflammation
Source: PLoS One. 2010 Aug 3;5(8):e11944. doi: 10.1371/journal.pone.0011944 (PMC2914748; doi:10.1371/journal.pone.0011944)

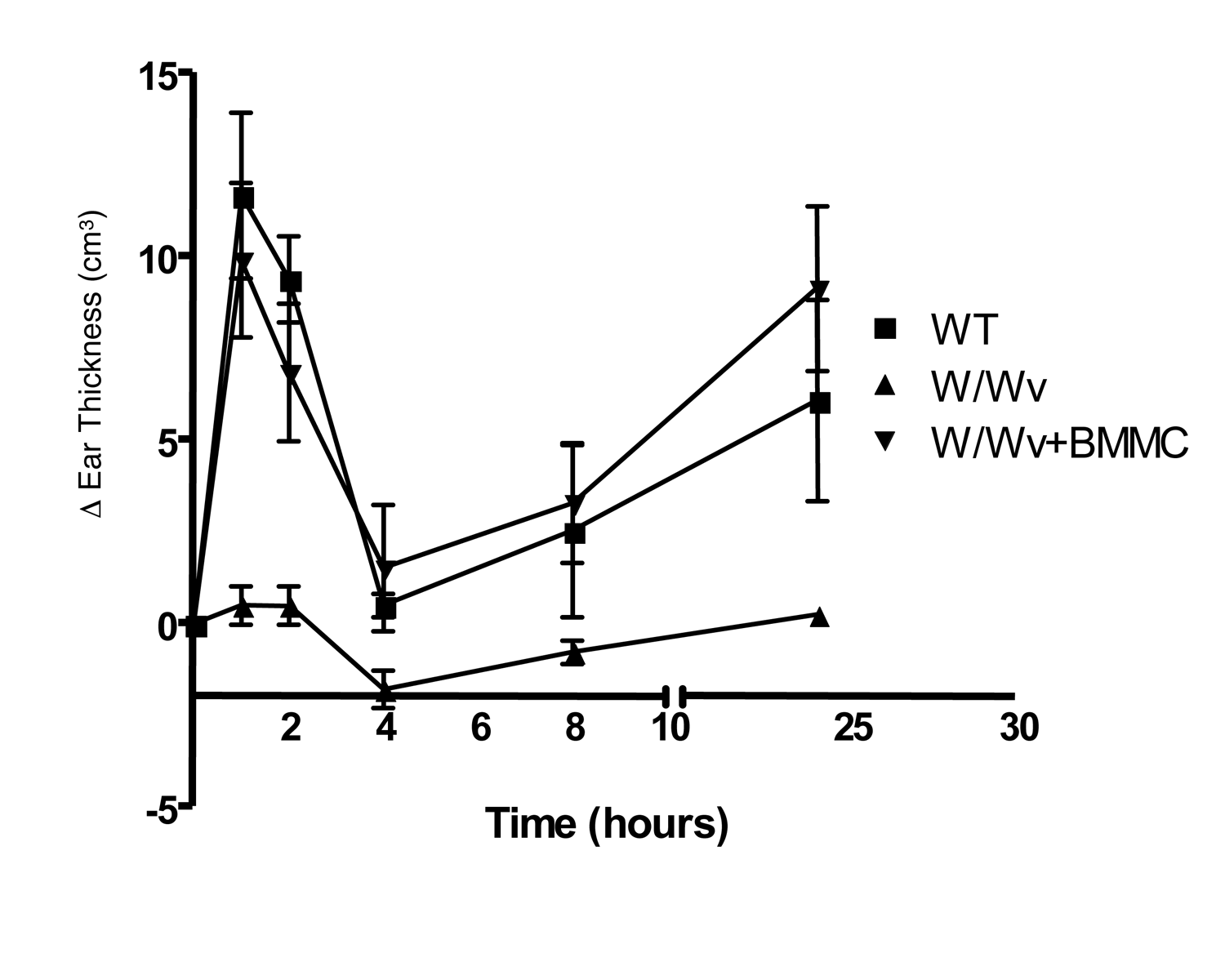

Supplement: Figure S1 — Passive cutaneous anaphylaxis is a mast cell-dependent model. Mast cells deficient (W/Wv) mice with or without reconstitution of mast cells with BMMC and littermates (WT) were investigated for their responses using the PCA model, as described in the methods. Ear swelling was measured at the indicated times. (0.14 MB TIF) [file pone.0011944.s001.tif]
